# Changes in Gene Expression of Pial Vessels of the Blood Brain Barrier during Murine Neurocysticercosis

**DOI:** 10.1371/journal.pntd.0002099

**Published:** 2013-03-14

**Authors:** Pramod Kumar Mishra, Judy M. Teale

**Affiliations:** 1 Department of Microbiology and Immunology, University of Texas Health Science Center, San Antonio, Texas, United States of America; 2 Department of Biology, University of Texas at San Antonio, San Antonio, Texas, United States of America; University of Edinburgh, United Kingdom

## Abstract

In murine neurocysticercosis (NCC), caused by infection with the parasite *Mesocestoides corti*, the breakdown of the Blood Brain Barrier (BBB) and associated leukocyte infiltration into the CNS is dependent on the anatomical location and type of vascular bed. Prior studies of NCC show that the BBB comprised of pial vessels are most affected in comparison to the BBB associated with the vasculature of other compartments, particularly parenchymal vessels. Herein, we describe a comprehensive study to characterize infection-induced changes in the genome wide gene expression of pial vessels using laser capture microdissection microscopy (LCM) combined with microarray analyses. Of the 380 genes that were found to be affected, 285 were upregulated and 95 were downregulated. Ingenuity Pathway Analysis (IPA) software was then used to assess the biological significance of differentially expressed genes. The most significantly affected networks of genes were “inflammatory response, cell-to-cell signaling and interaction, cellular movement”, “cellular movement, hematological system development and function, immune cell trafficking, and “antimicrobial response, cell-to-cell signaling and interaction embryonic development”. RT-PCR analyses validated the pattern of gene expression obtained from microarray analysis. In addition, chemokines CCL5 and CCL9 were confirmed at the protein level by immunofluorescence (IF) microscopy. Our data show altered gene expression related to immune and physiological functions and collectively provide insight into changes in BBB disruption and associated leukocyte infiltration during murine NCC.

## Author Summary

Neurocysticercosis (NCC) is one of the most common parasitic diseases of the CNS caused by the metacestode (larva) of the tapeworm *Taenia solium*. Epidemiological studies show that among the various forms of NCC, subarachnoid NCC is associated with poor prognosis, more resistance to anti-helminthic drugs and more severe inflammation. The chronic inflammation of the vasculature and arachnoid thickening (chronic basal meningitis) leads to blockade of CSF further contributing to CNS pathology. Using a murine model for NCC, we have found that among the different types of vasculature associated with the blood-brain barrier (BBB), pial vessels of BBB are compromised earlier and to a greater extent during NCC. In addition, pial vessels are likely the most important entryway for leukocyte infiltration during NCC. The aim of this study was to characterize infection-induced changes in the genome-wide gene expression of pial vessels. Our approach was to isolate pial vessels of the BBB by *in vivo* labeling of vessels followed by laser capture microdissection microscopy (LCM). Further, microarray analysis of pial vessels showed infection-induced changes in the expression of genes associated with both immunity and disease, and collectively provides insight into the dysfunction of the BBB and mechanisms associated with leukocyte infiltration during murine NCC.

## Introduction

The blood brain barrier (BBB) separates the peripheral circulation from the CNS and plays a critical role in homeostasis of the CNS environment. In the healthy brain BBB selectively restricts molecular and cellular trafficking between the blood and brain tissue and between blood and cerebrospinal fluid (CSF) [1]. The restrictive properties are largely controlled by specialized endothelial cells of the CNS vasculature which differ from those in the peripheral vasculature in terms of polarized expression of various transport systems, low transcytosis activity, high mitochondrial volume and sealing of the paracellular cleft between endothelial cells by continuous strands of interendothelial junction proteins including tight junctions [1]. However, additional components of the BBB are present in different CNS compartments and vary according to their anatomical location in the CNS and nature of the vasculature. The blood vessels present in leptomeninges (pia) in subarachnoid space are collectively termed pial vessels. The BBB associated with pial vessels in adult brain are largely devoid of pericytes, astrocytic endfeet processes, additional basement membranes and parenchymal tissue in comparison to that of parenchymal vessels [2], [3], [4]. Infection of the CNS leads to changes in barrier properties of the BBB allowing the leakage of serum components (edema) and infiltration of leukocytes resulting in CNS pathology [5], [6]. In addition, the BBB transport system is also affected further disturbing the homeostasis of the CNS environment [1].

Neurocysticercosis (NCC) is a CNS infection caused by the metacestode (larva) of the tapeworm *Taenia solium*. It is one of the most common parasitic infections of the CNS and a major cause of acquired epilepsy worldwide [7]. Depending upon the size, location, and number of parasites as well as sex, age and immune status of the host, there are differences in disease severity and pathologies [8]. Epidemiological studies show that among the various forms of NCC, subarachnoid NCC has the worst outcome and is associated with poor prognosis, more resistance to anti-helminthic drugs and more severe inflammation [9]. The chronic inflammation of the vasculature and arachnoid thickening (chronic basal meningitis) leads to blockade of CSF further contributing to CNS pathology [8].

Similarly, using a murine model for NCC by infection with the highly related parasite *Metacestoides corti*, prior studies from our laboratory have demonstrated that breakdown of the BBB and associated leukocyte infiltration depends on many criteria including the anatomical site, type of vascular bed, and infiltrating cell phenotype [6], [10], [11]. Assessment of the integrity of the BBB by changes in the architecture of interendothelial junction proteins and leakage of serum proteins revealed that the BBB associated with pial vessels were compromised earlier and to a greater extent in comparison to the BBB associated with vessels present in other CNS compartments [12], [13]. In addition, previous studies have shown that during murine NCC, the temporal pattern of infiltrating leukocyte subsets is characterized by a large infiltration of macrophages and γδ T cells followed by αβ T cells and lastly B cells [14]. Further characterization of leukocyte subset infiltration in different CNS compartments has established that the majority of the infiltration occurs via pial vessels [13].

There is a lack of detailed analysis of BBB disruption *in vivo* in a CNS compartment-specific manner. To address this deficiency and to obtain insights into changes occurring only to pial vessels, we designed a microarray-based, comprehensive study to analyze the changes in gene expression associated with the BBB comprised of pial vessels of the leptomeninges and subarachnoid spaces. We utilized laser capture microdissection microscopy (LCM) to isolate pial vessels from mock- and parasite-infected mice and performed microarray analyses. Our transcriptome data indicate an altered expression of genes related to the immune response and to physiological function and collectively provide insight into the dysfunction of the BBB during murine NCC associated with pial vessels.

## Materials and Methods

### Ethics statement

This study was conducted in strict accordance with the recommendations in the Guide for the Care and Use of Laboratory Animals of the U.S. National Institutes of Health. Experiments were carried out under the approved guidelines of the Institutional Animal Care and Use Committee (IACUC), University of Texas at San Antonio (approved IACUC protocol number MU003-07/11A0).

### Animals, parasites and infection

Female Balb/c mice were purchased from National Cancer Institute program (Bethesda, MD). Parasite maintenance and intracranial infection were performed using a protocol developed earlier [14]. *M. corti* metacestodes were maintained by serial intraperitoneal (i.p.) inoculation of 8- to 12-week-old female BALB/c mice. For intracranial inoculations, parasites were aseptically collected from the peritoneal cavity of mice that had been infected for about 4–6 months. Harvested parasites were extensively washed in HBSS. After that, the metacestodes (70 microorganisms) were suspended in 50 µl of HBSS and injected intracranially into 3–5-week-old female BALB/c mice using a 1-mL syringe and a 25-gauge needle using our protocol developed earlier. The needle was inserted to a 2-mm depth at the junction of the superior sagittal and the transverse sutures. This allows insertion of the needle into a protective cuff avoiding penetration of the brain tissue. Control mice were injected with 50 µl sterile HBSS using the same protocol. Before intracranial inoculation, mice were anesthetized intramuscularly with 50 µl mixture of ketamine HCL and xylazine (30 mg/ml ketamine and 4 mg/ml xylazine).

### *In vivo* labeling of vessels and laser captured microdissection

Animals were sacrificed at 3 weeks after inoculation. Before sacrifice, animals were anesthetized with 50 µl of mixture of ketamine HCL and xylazine. The thoracic cage was opened and 100–125 µl of a Rhodamine Red-X conjugated *Ricinus communis* agglutinin **(**Rh-RCA) lectin (Vector Lab) was injected through the left ventricle in heart. After 2 minutes of Rh-RCA injection, perfusion was performed through the left ventricle with 15 mL of cold HBSS [15]. Perfused brains were immediately removed, embedded in O.C.T. resin (Sakura, Torrance, CA) and snap frozen in 2-methyl butane (Fisher Scientific, Pittsburgh, PA) contained/cooled in liquid nitrogen and stored at −80°C for later use. 10 µm thick horizontal cryosections were obtained from each brain on polyethylene naphthalate membrane slides (Leica Microsystems, Wetzlar, Germany). The tissues were fixed in −20°C acetone for 20 seconds and kept in dry ice. Subsequently brain sections were dehydrated in 70% (10 s), 95% (20 s), 100% (3x, 30 s each) and xylene (2x, 30 sec). After dehydration, the slides were kept in desiccators until the time of dissection to avoid the humidity. LCM was performed with Leica LMD 7000 micro systems (Leica Microsystems, Wetzlar Germany) as described previously [16].

### RNA isolation and linear amplification

From LCM isolated endothelial cells, RNA was extracted with Pico Pure RNA isolation kit (Arcturus Bioscience, Mountain View, CA) according to manufacturer's protocol. DNase (Qiagen, Valencia, CA) treatment was performed directly within the purification column to remove any possible genomic contamination during the RNA extraction process. The quality of the RNA was inspected with Agilent 2100 Bioanalzyer and NanoDrop ND-1000. Samples passing quality control assessment were then subjected to linear amplification and subsequently labeled with NuGEN Ovation Aminoallyl RNA Amplification and Labeling System (NuGEN Technologies, San Carlos, CA) as per manufacturer's instructions.

### DNA microarray

Arrays were printed at the Duke Microarray Facility using the Genomics Solutions OmniGrid 100 Arrayer and mouse genome oligo set (version4.0). The *Mus musculus* Operon v4.0 spotted microarray contains 35,852 longmer probes representing 25,000 genes and about 38,000 gene transcripts (Operon Biotechnologies, Huntsville, AL).

### Microarray and data processing

The amplified and labeled product was hybridized to *Mus musculus* Operon v4.0 spotted microarray according to the manufacture protocol at 42°C with the MAUI hybridization system (BioMicro Systems, MAUI hybridization System, Salt Lake City, Utah). The array was then washed at increasing stringencies and scanned on a GenePix 4000B microarray scanner (Axon Instruments, Foster City, CA). The Genespring 11 program (Agilent Technologies, Redwood City, CA) was used to perform data processing and statistical analysis. Intensity-dependent (Lowess) normalization was done on the entire data set. To assess the quality of a data set, a principle component analysis was performed on samples on expression of all genes with mean centering and scaling. Datasets were filtered based on values and probe sets with background-subtracted intensity of 44 or less were excluded from the analysis. Subsequently, *t*-test analysis was performed to calculate the *p*-values using an asymptotic method and Benjamini-Hochberg, for multiple testing correction. Differentially expressed probe sets were selected based on volcano plot with a 2-fold change and *p*-value cut off of 0.05. Differentially expressed genes were then clustered using Average Linkage with Pearson Correlation as the similarity measurement. Molecular networks of the selected molecules and specific pathways were analyzed through Ingenuity Pathway Analysis software (Agilent Technologies, Redwood City, CA).

### Real time RT-PCR analysis

RNA obtained from LCM isolated endothelial cells (as described above) was subjected to linear amplification by the WT-Ovation Pico System (Nugen technology, San Carlos, CA). Resulting cDNA was loaded onto Taq-Man Low Density Arrays (Applied Biosystems, CA) microfluidic cards either preloaded with fluorogenic probes and custom-designed primers and housekeeping genes β-actin, ribosomal 18S, and GAPDH (glyceraldehyde 3-phosphate dehydrogenase) [17] or commercially available Mouse Immune Array (catalog number – 4367786, Applied Biosystems, CA). These plates were then loaded on an ABI Prism 7900 HT Sequence Detection System (Applied Biosystems, CA). The target expression levels were normalized to the levels of the house keeping genes 18S, β-actin and GAPDH in the same sample. Expression of each specific gene in infected samples over mock was calculated by ΔΔCt method and results are represented as ΔΔCt over mock [18].

### Tissue preparation and immunofluorescence microscopy

Tissue preparation and immunofluorescence (IF) staining was performed using our protocol as described previously [13]. Animals were sacrificed at 3 weeks after inoculation. Before sacrifice, animals were anesthetized with 50 µl of mouse cocktail and perfused through the left ventricle with 15 mL of cold PBS. Perfused brains were immediately removed, embedded in O.C.T. resin (Sakura, Torrance, CA) and stored at −80°C. Serial horizontal cryosections of 10 µm in thickness were placed on saline prep slides (Sigma-Aldrich, St. Louis, MO). The slides were air dried overnight and fixed in fresh acetone for 20 s at room temperature (rt). Acetone-fixed sections were wrapped in aluminum foil and stored at −80°C or processed immediately for immunofluorescence. Briefly, tissues were fixed in −20°C acetone for 10 min and then hydrated in PBS. Non-specific immunoglobulin binding was blocked by 30 min incubation at rt with 10% serum from the same species from which the fluorochrome conjugated antibodies (secondary antibodies) were derived. Sections were incubated for 40 min with primary antibodies diluted in 3% serum from the host of secondary antibody. Sections were washed 7× for 3 min each after incubation with specified antibodies. Secondary antibodies were incubated for 30 min at rt when necessary. Then, sections were mounted using fluorsave reagent (Calbiochem, La Jolla, CA) containing 0.3 µM 4′,6′-diamidino-2-phenylindole dilactate-DAPI (Molecular Probes, Eugene, OR). Negative controls using secondary antibodies alone were included in each experiment and found to be negative for staining. Fluorescence was visualized in a Leica microscope (Leica Microsystems, Wetzlar Germany). Images were acquired and processed using IP lab software (Scanalytics, Inc., Rockville, MD, USA) and Adobe Photoshop CS2 (Adobe, Mountain View, CA). The purified primary antibodies goat anti mouse CCL5 (catalog number AF478) and CCL9 (catalog number AF463) were bought from R&D systems and biotinylated CD31 antibody (catalog number 553371) from Pharmingen (San Diego, CA). Rabbit anti Goat labeled with Rhodamine Red- X and donkey anti rabbit rhodamine red X secondary antibodies were purchased from Jackson ImmunoResearch (West Grove, PA) [13].

### *M. corti* supernatant and homogenate preparation

*M. corti* parasites were collected aseptically from 4–6 months ip infected mice and washed rigorously with HBSS and then incubated with half the volume of HBSS+ gentamycin at 37°C, 4% CO_2_ for 72 hrs in a 25 CM^2^ culture flask. After incubation, parasites were removed by filtering with a nylon mesh and the supernatant (MCS) was collected and kept at −80°C for future use.

### Endothelial cell culture and immunofluorescence

bEND.3 cells were purchased from ATCC and subcultured using DMEM+10%FBS. Cell were seeded in chamber slides and stimulated with parasite supernatant, parasite homogenate or PBS for control. After, 72 hrs of stimulation, IF staining was performed. Briefly, cells were washed with PBS and incubated with 70% ETOH for 10 minutes followed by 3 PBS washes for 3 min each. Subsequently, cells were blocked with 10% serum from the host of secondary antibody, followed by 40 min incubation with primary antibodies and 30 min with secondary antibodies as described in previous (IF section) section. Chamber slides were mounted using fluorsave reagent (Calbiochem, La Jolla, CA) containing DAPI. Images were acquired and processed as described in the previous section.

## Results

### *In vivo* labeling of BBB and LCM

**Figure 1:**
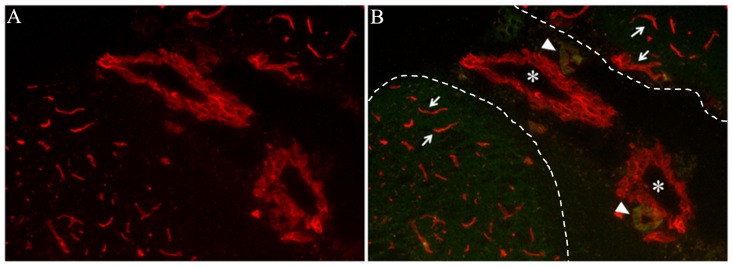
*In vivo* labeling of blood vessels with Rh-RCA. Representative Images (40X) of dehydrated brain section showing differential labeling of BBB by *Ricinus communis* agglutinin (RCA) lectin conjugated with rhodamine dye (Rh-RCA). (A) *In vivo* labeled pial vessels (red) in subarachnoid space. (B) Labeled vessels (red) with background (green) to differentiate between venules (asterisks) and arterioles (arrowheads) of pial vessels of BBB in the leptomeninges under the pia (dotted lines) and parenchymal vessels of BBB (arrows) in parenchyma.

We administered Rh-RCA lectin (Rhodamine conjugated *Ricinus communis* agglutinin**)** systemically at 3 wk post infection (p.i.) and mock-infected mice to label the pial vessels as described in Materials and Methods. The 3 wk p.i. time point was used because this is consistently the peak of leukocyte infiltration. Brain sections from *in vivo* labeled, perfused brain tissues were prepared and analyzed for labeling of the blood vessels after dehydration. We found that 5 µg/mg of body weight was sufficient to label the blood vessels (Fig. 1). LCM was performed as described previously [16]. RH-RCA labeled Pial vessels, distinctly located in subarachnoid spaces along with leptomeninges were collected by LCM. Subsequently, total RNA was isolated from LCM enabled samples, and linear amplification was done in order to perform microarray experiments as described in Material and Methods.

### Identification of differentially expressed genes

**Figure 2:**
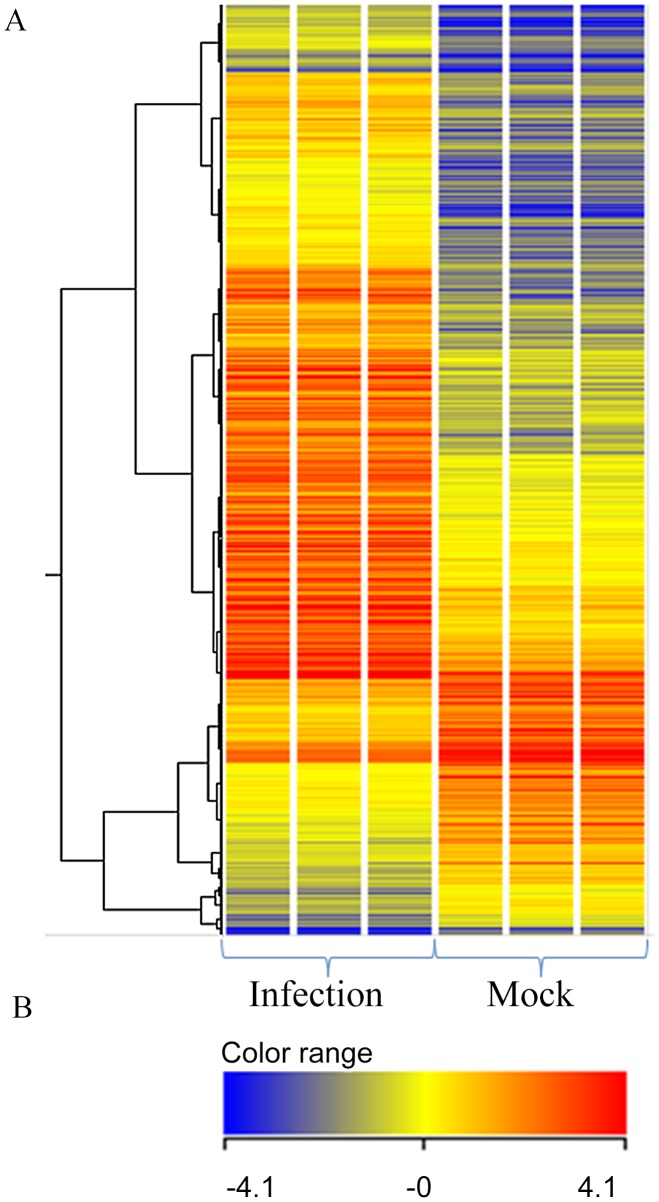
Hierarchical cluster analysis of differentially expressed probe sets in pial endothelial cells. First 3 columns represent 3 biological replicates of 3 wks p.i. mice whereas last 3 columns represent 3 independent mock-infected samples (A). A bar showing the color range used for denoting the upregulated and downregulated genes (B).

Microarray hybridization experiments were performed to assess differentially expressed genes during infection using operon spotted chip arrays, and the data were processed by Genespring 11 to quantify differentially expressed probe sets (see Materials and Methods). Quality control on samples was done by principle component analysis which showed separation between mock and infected samples based on their gene expression profile while clustering the infected samples and mock samples together respectively (data not shown). In total, 2154 probe sets passed the screen when the probe sets were filtered for intensity with a lower cut off 44. Out of these, 768 probe sets met a corrected p-value (Benjamini-Hochberg cut off of 0.05. Of the 768 probe sets, 578 probe sets were found to be differentially expressed with a fold change of ≥2. Differentially expressed probe sets with a fold change of ≥2 were subjected to hierarchical cluster analysis using Average Linkage with Pearson Correlation as the similarity measurement of gene expression (Fig. 2).

Operon chips contain oligo probe sets representing transcripts belonging to annotated genes as well as Expression Sequence Tags (EST) which represent yet to be defined genes. All the 578 differentially expressed probes were uploaded to Ingenuity Pathway Analysis (IPA) software to find out known genes associated with differentially expressed probe sets. IPA is a web-based application that uses a knowledge base created by previous findings of molecular interactions in the context of biological events. Once a gene is uploaded into IPA during core analysis, it maps the gene and places them in relevant molecular networks, biofunctions and specific pathways (https://analysis.ingenuity.com/). Out of 578 probe sets, 380 (285 upregulated and 95 down regulated) were found annotated or mapped by IPA (Table S1).

### Assessment of biological significance of differentially expressed genes of pial endothelium

In order to understand the biological significance of the differentially expressed genes, biofunctions and networks of genes involved in biofunctions were analyzed using IPA. Under biofunction analysis genes were categorized into three different classes of biofunctions such as disease and disorder, molecular and cellular function, and physiological system development and function (Table 1). The disease and disorder category included Immunological disease, infectious disease, inflammatory response, connective tissue disorders and inflammatory disease (p = 8.17E-24 to 2.83E-05). The category of molecular and cellular functions included Cellular function and maintenance, cellular movement, cell death, cellular development and cellular growth and proliferation (p = 1.00E-33 to 3.92E-05). Genes in the category of physiological system development and function were associated with Hematological system development and function, tissue morphology, immune cell trafficking, tissue development and humoral immune response (p = 9.38E-32 to3.87E-05) (Table 1).

**Table 1:** Top biological functions associated with differentially expressed genes.

Name	p-value	# Molecules
**Diseases and disorders**		
Immunological disease	8.17E-24 - 5.07E-05	119
Infectious disease	1.17E-23 - 5.16E-05	78
Inflammatory response	4.87E-22 - 3.87E-05	142
Connective tissue disorders	7.08E-19 - 1.22E-06	82
Inflammatory disease	7.08E-19 - 2.83E-05	109
**Molecular and cellular functions**		
Cellular function and maintenance	1.00E-33 - 2.83E-05	127
Cellular movement	9.93E-28 - 4.60E-05	119
Cell death	2.42E-23 - 5.20E-05	161
Cellular development	1.52E-21 - 4.60E-05	152
Cellular growth and proliferation	1.52E-21 - 3.92E-05	166
**Physiological system development and function**		
Hematological system development and function	9.38E-32 - 4.85E-05	152
Tissue morphology	9.38E-32 - 4.85E-05	121
Immune cell trafficking	9.93E-28 - 3.87E-05	105
Tissue development	5.03E-18 - 4.85E-05	104
Humoral immune response	6.19E-16 - 3.87E-05	59

**Figure 3:**
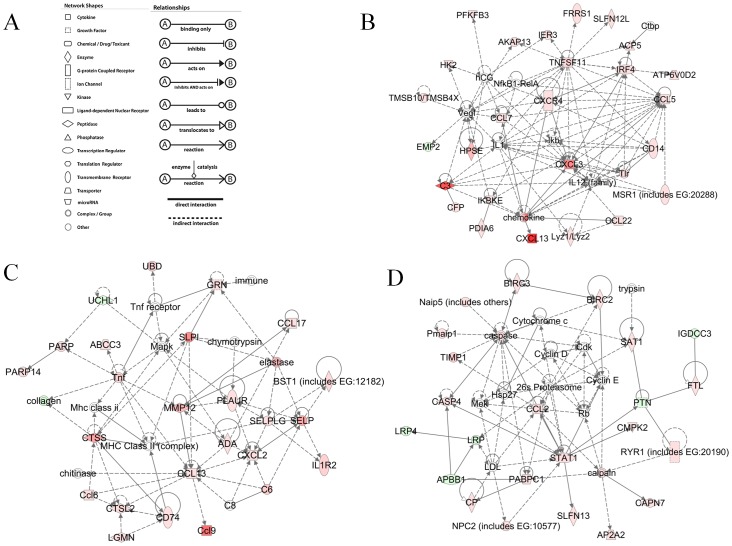
Schematic representation of the significant networks containing immune response genes. (A) Shape and relation legend. (B) Inflammatory response, cell-to-cell signaling and interaction, cellular movement. (C) Cellular movement, hematological system development and function, immune cell trafficking. (D) Antimicrobial response, cell-to-cell signaling and interaction, embryonic development. Green color represents down regulated genes, red color represents upregulated genes and genes without color are not affected in the data set but are relevant for the generation of the networks.

Many of the genes were classified in more than one biofunction category due to the broad and overlapping nature of the categories as well as an individual gene influencing multiple biofunctions. We analyzed the differentially expressed genes using IPA to assess how genes interact with each other as part of biological pathways. The resulting networks are generated based on the random selection of focus genes with maximum connectivity and several interconnected focus genes put together as a network in order of high to low scores. Scores are derived from p-values and are calculated through Fisher's exact test which represents the probability of finding the focus genes of a network in a set of n genes randomly selected from a global molecular network of genes. Based on focus genes differentially expressed during infection, 23 networks were identified. 22 networks that yielded a score of more than 3 are shown in Table S2. Network analysis indicated that genes involved in the metabolism of lipids, carbohydrates and amino acids are affected. Further, immune response related genes were identified in multiple networks along with genes involved in cell growth, death and connective tissue disorder (Table S2). Pictorial representation of three of the networks is shown in Fig. 3. Fig. 3 B, C and D show the networks “inflammatory response, cell-to-cell signaling and interaction, cellular movement” “cellular movement, hematological system development and function, immune cell trafficking” and “antimicrobial response, cell-to-cell signaling and interaction, embryonic development” respectively involving immune response related genes.

### Validation of microarray results

A number of genes were chosen from different functional categories to be verified for their gene expression pattern by Taqman real time polymerase chain reaction (RT-PCR) using the amplified cDNA derived from pial endothelial cells isolated by LCM. Results obtained from RT-PCR experiments confirmed the expression pattern of a number of genes. Data showed that SELP, CD274, LGALS3, MRC1, FIZZ1, β2M, C3, CCL2, CCL5 and STAT1 were significantly upregulated (Table 2) similar to microarray.

**Table 2:** Validation for gene expression pattern by RT-PCR.

Gene	Accession no	Function	Microarray fold change	RT-PCR		
				avg ΔΔCt	std error	P value
SELP	NM_011347	Adhesion	31.48	5.61	1.2	<0.001
CCL2	NM_011333	Chemotaxis	10.95	5.78	0.54	<0.001
CCL5	NM_013653	Chemotaxis	7.47	4.36	0.50	<0.001
FIZZ1	NM_020509	Tissue remodelling	8.51	10.61	0.2	<0.001
LGALS3	NM_010705	Adhesion and chemotaxis	14.94	5.43	0.5	<0.001
MRC1	NM_008625	Phagocytosis	5.62	5.19	0.9	<0.05
β2M	NM_009735	Antigen presentation	4.79	3.10	0.4	<0.01
C3	NM_009778	Inflammation and migration	61.38	8.81	1.26	<0.01
STAT1	NM_011487	Signaling molecule	2.66	2.94	0.60	<0.01

**Figure 4:**
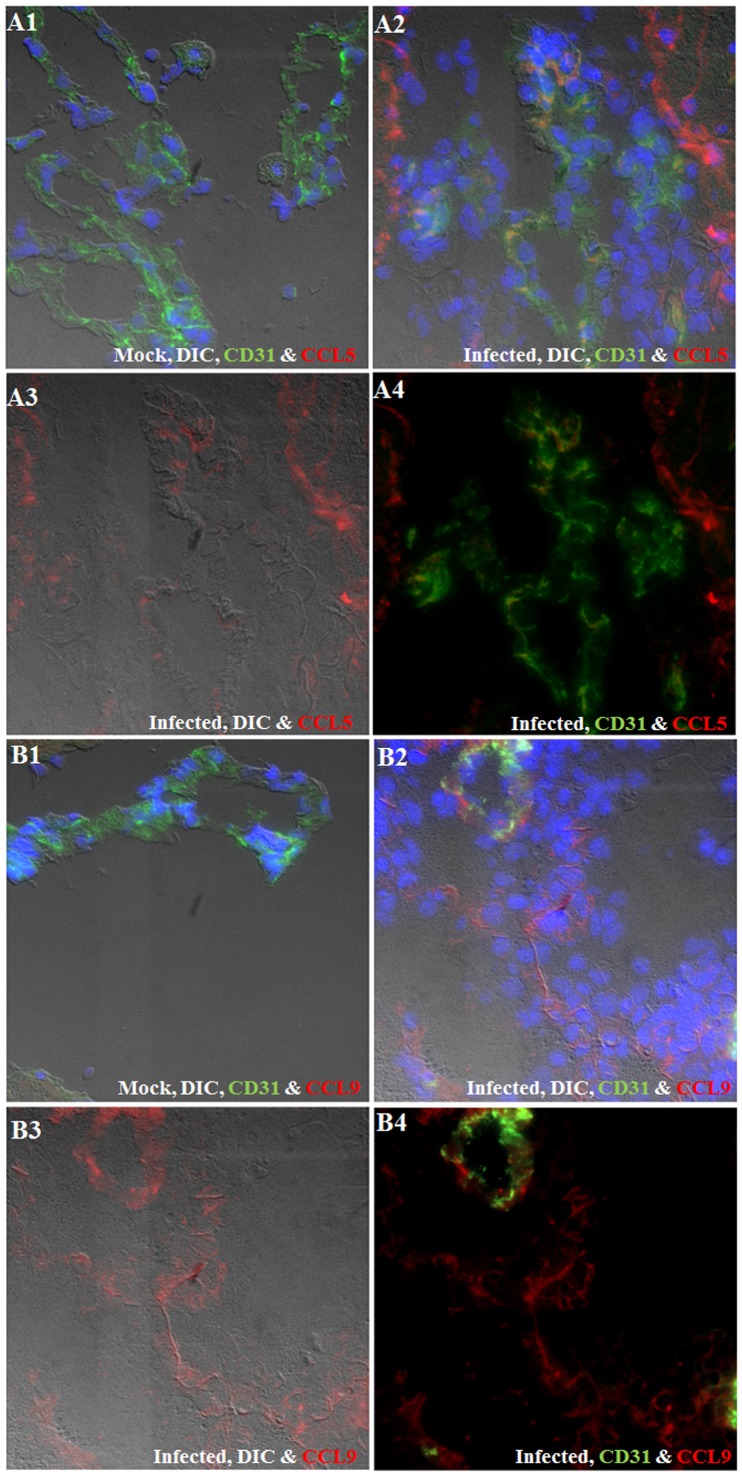
Immunofluorescence staining of cryosections obtained from mock-infected and NCC brain samples for chemokines . Immunofluorescence staining (IF) of cryosections obtained from mock-infected (A1 & B1) and 3 wks p.i. NCC brain (A2–A4 & B2–B4) sections. Chemokine CCL5, CCL9 are shown in red color, CD31, an endothelial cell marker is shown in green color and DAPI representing nuclear staining is shown in blue color. (**A1–A4**) CCL5 expression in endothelial cells (A1) Mock sample (IF+DIC, 40 X). (A2) Infected sample (IF+DIC, 40x). (A3) CCL5 IF with DIC. (A4) CCL5 with CD31 IF. (**B1–B4**) CCL9 expression in endothelial cells. (B1) Mock (IF+DIC, 40 X). (B2) Infected sample (IF+DIC, 40x). (B3) CCL9 IF with DIC. (B4) CCL9 with CD31 IF.

To assess protein expression, brain sections from mock-infected and infected mice (3 wk p.i.) were analyzed by IF microscopy for chemokines including CCL5 (Fig. 4A) and CCL9 (Fig. 4B). In sections from mock-infected mice, CCL5 was undetectable. Infection resulted in a substantial up-regulation of CCL5 which co-localized with CD31, an endothelial cell marker. Similarly, CCL9 was scarcely detected in the blood vessels from mock-infected samples. CCL9 was highly up-regulated as a result of infection and appears to be secreted. In addition, it co-localizes with undefined strand-like structures that appear to form a gradient starting from the outer surface of pial vessels (abluminal) towards the direction of infiltrating cells. The degree of CCL9 expression was higher in inflamed vessels exhibiting leukocyte egress.

**Figure 5:**
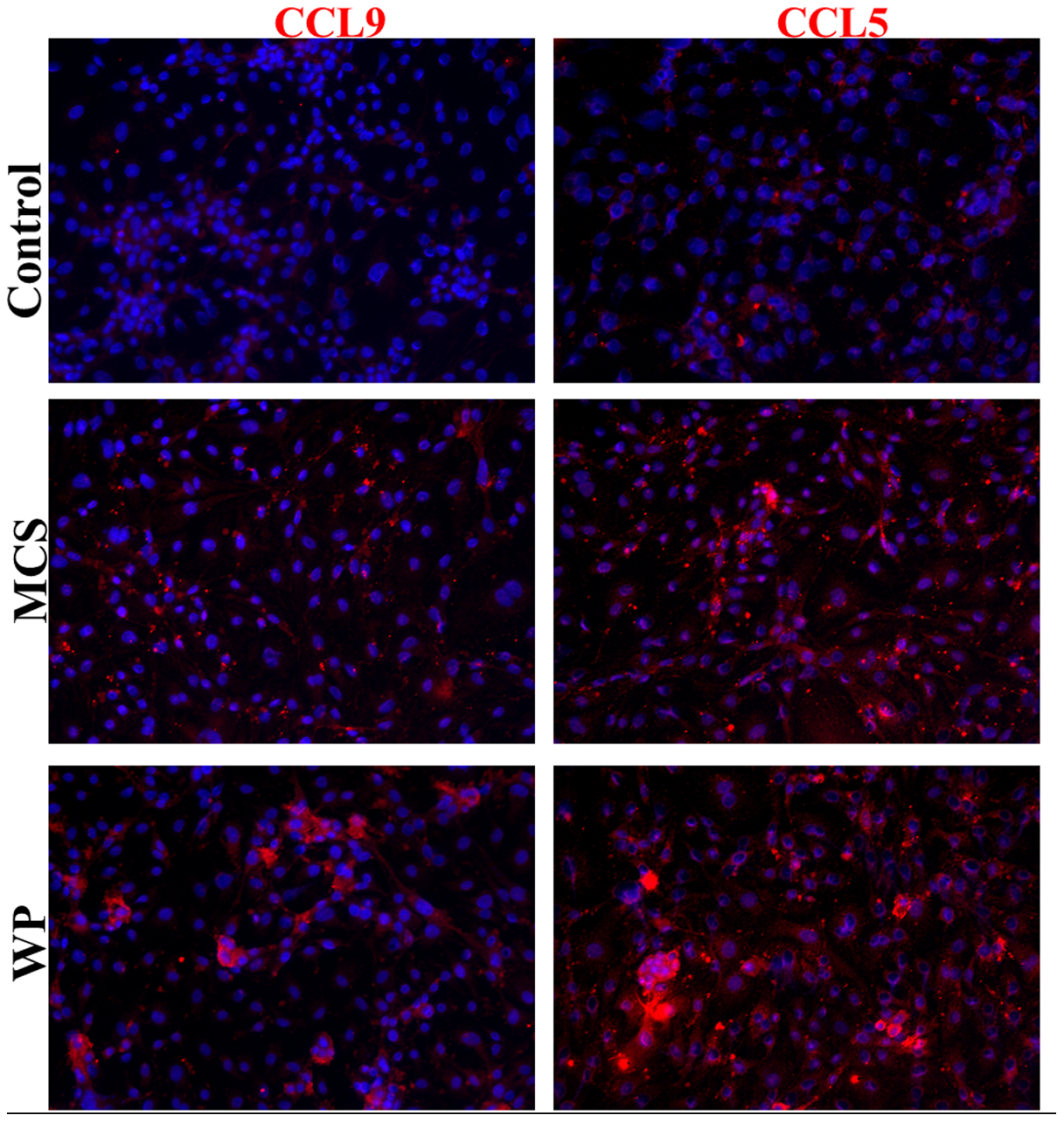
Immunofluorescence staining of bEND.3 cells showing the expression of CCL5 and CCL9. bEND.3 cells (endothelial cells) stimulated with parasite supernatant (MCS), parasite homogenate (WP) or PBS for control for 72 hrs. Representative 20X images of control, MCS and WP stimulated bEND.3 cells showing induction of CCL5 and CCL9 are shown in red color while blue color represent DAPI staining of nuclei.

Since chemokines can be secreted and deposited on extracellular matrix, it was important to confirm that endothelial cells can produce these chemokines. To test this, bEND.3 (brain endothelial cell line) cells were stimulated with either *M. corti* secretory/released antigens (MCS) or whole parasite homogenate in HBSS (WP) and analyzed for the production of CCL5 and CCL9. We found that both parasite preparations induced an increased expression of CCL5 and CCL9 by bEND.3 cells compared with controls in the absence of antigen (Fig. 5).

## Discussion

The BBB acts as an interface between the periphery and the CNS and tightly regulates the components of the immune response to prevent unnecessary inflammation/pathology in the healthy brain. It is known that the nature of the vasculature and associated functions differ greatly depending upon their location in different CNS compartments [4]. In a number of CNS infections, pial vessels of the BBB are particularly prone to disruption with leakage of leukocytes and serum components leading to meningitis [19]. This increased vulnerability is possibly due to lack of additional barrier components and potential exposure to antigens compared with parenchymal vessels [20], [21], [22]. Previously, gene expression analysis of endothelial cells has been performed either in an *in vitro* setting or with whole brain endothelial cells [23], [24], [25], [26], [27], but not with endothelial cells present in specific anatomical compartments. Moreover, the effect of parasitic infection on endothelial cell biology has not been studied. The focus of this study was to characterize the infection-induced molecular signature of LCM isolated pial endothelial cells by evaluating global gene expression by microarray analyses.

LCM allowed us to isolate cells present in a specific location which has an added advantage over other marker-based techniques such as FACS. However, one pitfall is that the potential contamination of the BBB endothelium with the leukocyte that may be extravasating or adhering to the endothelial cells. Our data analysis confirmed that differential gene expression data obtained through microarray hybridization experiment is mainly contributed by endothelial cells comprising the BBB as common lymphoid or myeloid cell markers were not detectable in the data set. In addition, the expression of the following BBB specific transporter markers were induced during infection: TFRC (related to iron metabolism), ABCG1 (cholesterol homeostasis), SLC15A3 (proton oligopeptide co-transporters), SLC7A5 (cationic amino acid transporters and the glycoprotein-associated amino acid transporters), ABCC3 (multidrug resistance associated protein 3) and ABCC5 (multidrug resistance associated protein 5). Other BBB specific markers were downregulated including SLC9A3R2 (sodium/hydrogen exchanger), SLC6A9 (neurotransmitter transporter, glycine, sodium and chloride dependent neurotransmitter) [23], [24], [25], [26], [27].

Network analysis shows that apart from transporters several other sets of immune related genes including MRC1, complements (C3, C6, and C1R and complement factor properdin), TNF super family members and interferon inducible genes including STAT1 are induced in NCC infection which can potentially lead to endothelial cell activation [23], [24], [28]. Interferon inducible genes have been shown to be induced in an *in vitro* study with endothelial cells in HIV and *Cryptococcus neoformans* infection model [25], [29]. STAT1 has been shown to promote inflammatory mediators and leukocyte transmigration at the BBB [30]. Interferon signaling mediated through the Jak Stat pathway is critical to induce several of these genes in endothelial cells including chemokines and MHC class I antigen presentation related genes [23], [24], [28].

Among immune related genes chemokines play a critical role in leukocyte trafficking, differentiation and angiogenesis or angiostasis [31], [32]. Leukocyte trafficking is a multistep process in which chemokines induce the migration of leukocytes toward a chemokine gradient. Interaction between chemokines expressed by endothelial cells with their receptors on leukocytes triggers a signaling process that increases the avidity of integrin to their receptors on endothelial cells causing firm adhesion of leukocytes and facilitated transmigration towards chemokine gradient [33]. Chemokines are divided into C, CC, CXC, and CX3C subgroups based on conserved cysteine residues [31]. The present study advances the understanding about chemokine expression profile in endothelial cells comprising the BBB which are the first CNS cells to encounter peripheral leukocytes *in vivo*. Many of the chemokines upregulated (Table S1) in response to infection are summarized in Table 3 along with their putative receptor and influence on specific leukocyte subsets.

**Table 3:** Parasite infection induced chemokines in pial endothelial cells and their known role in leukocyte trafficking.

Chemokines	Receptors	Chemotaxis for	Functional Implication Based on Literature
CXCL2 CXCL3	CXCR2	Granulocytes/neutrophils	Impaired neutrophil extravasation in CXCR2^−/−^ mice in experimental brain abscesses model [39]. Impaired neutrophil recruitment in CXCR2^−/−^ mice during river blindness [40]. Attenuation of neutrophil infiltration in CXCR2^−/−^ mice during head injury [41]. CXCL2 induced P-selectin-dependent neutrophil rolling and extravascular migration *in vivo* [42]
CXCL13	CXCR5 [43]	B Cells [43], T cells [44], CD3^+^CD4^−^CD8^−^ double negative (DN) T cells [45], Treg [46]	Lymphoneogenesis [44].CD4^+^ (follicular) T cells [44], [47]. CXCL13 expression correlated with increased frequency of B cells and CXCR5^+^T (≈20%) cells in the CSF of MS patients [44]. Antibody neutralization led to reduced B cell chemotaxis during neuroboreliosis [44]. CXCL13 antibody neutralization abrogated DN T regs [45]
CCL2	CCR2	Inflammatory monocytes [48], [49], [50], [51]	CCL2^−/−^ mice had defect in recruitment of monocytes in CNS in EAE [48]. CCR2^−/−^ failed to recruit monocytes during EAE [49], [50]
CCL5	CCR1, CCR3, CCR5 [52]	γδ T cells [53], αβ T cells (Th1) [52], macrophage [54]	CCL5 neutralization led to reduced leukocyte in CNS in EAE. CCR5^−/−^ mice had reduced number of CD4, CD8 and macrophage in west Nile Virus infection [54]. Antibody neutralization of CCL5 or CCR5 inhibited transmigration of Th1 cells [52]
CCL6	CCR1	Macrophage [55], [56]	
CCL9	CCR1, CXCR3	Immature myeloid cells (iMC) [57], CD11b+ dendritic cells (DCs) [58]	CCL9 down modulation by shRNA in cancer cells correlated with reduction in iMC [57]. Antibody blocking of CCL9 resulted in reduction of CD11b^+^DC [58]
CCL17, CCL22	CCR4	αβ T cells (Th2) [59]	Monoclonal antibody against CCR4 reduced chemotaxis in response to MDC (CCL22)/TARC (CCL17) [59]

Our *in vivo* and *in vitro* data shows that CCL9 is expressed abundantly by endothelial cells and appears to coat the strands in a gradient fashion. Such strands have been observed in the areas of inflammation in other disease conditions such as EAE and toxoplasmic encephalitis [34]. The origin and composition of these strands are still not clear. They have been described to extend from blood vessels to parenchyma and are thought to provide structural support for leukocytes migration [34]. In the case of NCC, these strands coated with CCL9 might also provide a physical scaffold structure with a chemotactic signal for migration of leukocytes into the CNS. The functional correlation for CCL9 in terms of leukocyte subset recruitment remains to be defined in the CNS. However, in the periphery CCL9 has been implicated in recruitment of myeloid cells to peyers' patches and osteoclasts through the CCR1 receptor. Furthermore, it is also critical to recruit immature myeloid cells through CCR1receptor during liver metastasis [35]. In addition, CCL17 and CCL22 are also noteworthy as they have been implicated in trafficking of CCR4 positive regulatory and Th2 T cells subsets [33]. Chemokine can selectively influence the trafficking of leukocyte subsets. Therefore, the expression profile of chemokines in the BBB provides insight into the trafficking of different leukocyte subsets such as M1 and M2 macrophages, granulocytes, γδ T cells, αβ T cells and B cells known to infiltrate during NCC [13], [14], [36], [37], [38].

In summary, our data delineate infection-induced changes in the expression of genes associated with both immunity and disease, and collectively provide insight into the dysfunction of the BBB and mechanisms associated with leukocyte infiltration during murine NCC.

## Supporting Information

Table S1 **Differentially expressed genes in pial endothelial cells of BBB.** Column one shows the fold change from most upregulated to most downregulated genes, column 2 shows the genebank id, column 3 shows gene symbol and column 4 shows entrez gene name.
(PDF)

Table S2 **List of significant networks of genes.** Networks associated with differentially expressed genes in pial endothelial cells containing down regulated genes shown by green color, upregulated genes shown by red color and genes which are not affected in endothelial cells during infection but relevant for the generation of the networks are shown in black color.
(PDF)
