# Supplementary material for: Changes in Gene Expression of Pial Vessels of the Blood Brain Barrier during Murine Neurocysticercosis
Source: PLoS Negl Trop Dis. 2013 Mar 14;7(3):e2099. doi: 10.1371/journal.pntd.0002099 (PMC3597490; doi:10.1371/journal.pntd.0002099)
Supplement: Table S1 — Differentially expressed genes in pial endothelial cells of BBB. Column one shows the fold change from most upregulated to most downregulated genes, column 2 shows the genebank id, column 3 shows gene symbol and column 4 shows entrez gene name. (PDF) [file pntd.0002099.s001.pdf]

Fold Change	ID	Symbol	Entrez Gene Name
80.621	NM_018866	CXCL13	chemokine (C-X-C motif) ligand 13
73.072	NM_021396	PDCD1LG2 (includes EG:309304)	programmed cell death 1 ligand 2
64.686	NM_011315	SAA1	serum amyloid A1
61.387	NM_009778	C3	complement component 3
51.824	NM_011338	Ccl9	chemokine (C-C motif) ligand 9
48.334	NM_007482	ARG1	arginase, liver
42.102	NM_009140	CXCL3	chemokine (C-X-C motif) ligand 3
41.919	NM_172715	AGPAT9	1-acylglycerol-3-phosphate O-acyltransferase 9
40.475	NM_010196	FGA	fibrinogen alpha chain
40.377	NM_011414	SLPI	secretory leukocyte peptidase inhibitor
32.886	NM_025658	Ms4a4b (includes others)	membrane-spanning 4-domains, subfamily A, member 4B
32.086	NM_021281	CTSS	cathepsin S
31.488	NM_011347	SELP	selectin P (granule membrane protein 140kDa, antigen CD62)
25.164	NM_007449	Ang2 (includes	angiogenin, ribonuclease A family, member 2
24.167	NM_152803	HPSE	heparanase
23.807	NM_008605	MMP12	matrix metallopeptidase 12 (macrophage elastase)
23.156	NM_008329	Ifi204 (includes	interferon activated gene 204
23.062	NM_011410	SLFN12	schlafen family member 12
20.644	NM_008147	Gp49a/Lilrb4	leukocyte immunoglobulin-like receptor, subfamily B, member 4
19.073	NM_139198	PLAC8	placenta-specific 8
18.425	NM_009853	CD68	CD68 molecule
18.192	NM_016704	C6	complement component 6
17.167	NM_011593	TIMP1	TIMP metallopeptidase inhibitor 1
17.101	NM_018746	ITIH4	inter-alpha-trypsin inhibitor heavy chain family, member 4
16.437	NM_010555	IL1R2	interleukin 1 receptor, type II
16.070	NM_010401	HAL	histidine ammonia-lyase
15.893	NM_021394	ZBP1	Z-DNA binding protein 1
15.415	NM_023137	UBD	ubiquitin D
14.942	NM_010705	LGALS3	lectin, galactoside-binding, soluble, 3
14.903	NM_011613	TNFSF11	tumor necrosis factor (ligand) superfamily, member 11
14.801	NM_053110	GPNMB	glycoprotein (transmembrane) nmb
14.589	NM_173394	TICAM2	toll-like receptor adaptor molecule 2
13.489	NM_009763	BST1 (includes EG:12182)	bone marrow stromal cell antigen 1

13.314	NM_022430	MS4A8B	membrane-spanning 4-domains, subfamily A, member 8B
12.888	NM_010187	FCGR2B	Fc fragment of IgG, low affinity IIb, receptor (CD32)
12.756	NM_009827	CCKAR	cholecystokinin A receptor
12.531	NM_019549	PLEK	pleckstrin
12.225	NM_008842	PIM1	pim-1 oncogene
12.156	NM_009117	SAA2	serum amyloid A2
11.851	NM_009984	CTSL2	cathepsin L2
11.617	NM_182806	GPR18	G protein-coupled receptor 18
11.421	NM_011175	LGMN	legumain
11.322	NM_175026	BC094916/Pyhin1	pyrin and HIN domain family, member 1
11.275	NM_033584	PCDHGA1	protocadherin gamma subfamily A, 1
11.086	XM_128954	TMEM173	transmembrane protein 173
10.627	NM_011333	CCL13	chemokine (C-C motif) ligand 13
10.562	NM_009139	Ccl6	chemokine (C-C motif) ligand 6
10.512	NM_011408	SLFN12L	schlafen family member 12-like
10.443	NM_134152	LPXN	leupaxin
10.270	NM_172451	GALNT6	UDP-N-acetyl-alpha-D-galactosamine:polypeptide N-acetylgalactosaminyltransferase 6 (GalNAc-T6)
10.250	NM_010442	HMOX1	heme oxygenase (decycling) 1
9.920	NM_009593	ABCG1	ATP-binding cassette, sub-family G (WHITE), member 1
9.799	NM_031195	MSR1 (includes EG:20288)	macrophage scavenger receptor 1
9.605	NM_031254	TREM2	triggering receptor expressed on myeloid cells 2
9.534	NM_007398	ADA	adenosine deaminase
9.459	NM_011227	RAB20	RAB20, member RAS oncogene family
9.419	NM_013654	CCL7	chemokine (C-C motif) ligand 7
9.285	NM_008176	CXCL2	chemokine (C-X-C motif) ligand 2
9.272	NM_007995	Fcna	ficolin A
9.179	NM_011426	SIGLEC1	sialic acid binding Ig-like lectin 1, sialoadhesin
9.175	NM_011061	PADI4	peptidyl arginine deiminase, type IV
9.164	NM_023044	SLC15A3	solute carrier family 15, member 3
9.104	NM_139200	CYTIP	cytohesin 1 interacting protein
8.845	NM_008327	Ifi202b	interferon activated gene 202B
8.668	NM_175406	ATP6V0D2	ATPase, H+ transporting, lysosomal 38kDa, V0 subunit d2
8.618	NM_015811	RGS1	regulator of G-protein signaling 1
8.610	NM_011827	HCST	hematopoietic cell signal transducer
8.591	NM_011331	CCL2	chemokine (C-C motif) ligand 2
8.526	NM_021451	Pmaip1	phorbol-12-myristate-13-acetate-induced protein 1
8.515	NM_020509	Retnla	resistin like alpha

8.460	NM_030710	SLAMF6	SLAM family member 6
8.424	NM_028773	SASH3	SAM and SH3 domain containing 3
8.391	NM_145209	OASL	2'-5'-oligoadenylate synthetase-like
8.337	NM_011260	REG3A	regenerating islet-derived 3 alpha
8.110	NM_022325	CTSZ	cathepsin Z
8.075	NM_010185	FCER1G	Fc fragment of IgE, high affinity I, receptor for; gamma polypeptide
8.021	NM_011407	Slfn1	schlafen 1
7.951	XM_195661	B3GNT6	UDP-GlcNAc:betaGal beta-1,3-N-acetylglucosaminyltransferase 6 (core 3 synthase)
7.935	NM_018851	SAMHD1	SAM domain and HD domain 1
7.851	NM_145227	OAS2	2'-5'-oligoadenylate synthetase 2, 69/71kDa
7.785	NM_178611	LAIR1	leukocyte-associated immunoglobulin-like receptor 1
7.784	NM_031367	IFI44L	interferon-induced protein 44-like
7.740	NM_011691	VAV1	vav 1 guanine nucleotide exchange factor
7.708	NM_134066	AKR1C3	aldo-keto reductase family 1, member C3 (3-alpha hydroxysteroid dehydrogenase, type II)
7.679	NM_021893	CD274	CD274 molecule
7.617	NM_009911	CXCR4	chemokine (C-X-C motif) receptor 4
7.475	NM_013653	CCL5	chemokine (C-C motif) ligand 5
7.450	NM_007707	SOCS3	suppressor of cytokine signaling 3
7.438	NM_007388	ACP5	acid phosphatase 5, tartrate resistant
7.355	NM_009977	CST7	cystatin F (leukocystatin)
7.330	NM_011157	SRGN	serglycin
7.271	NM_021792	Iigp1/Iigp1b	interferon inducible GTPase 1
7.255	NM_027123	FASTKD3	FAST kinase domains 3
7.192	NM_011638	TFRC	transferrin receptor (p90, CD71)
7.122	NM_013706	CD52	CD52 molecule
7.064	XM_135820	CFP	complement factor properdin
7.001	XM_134619	MMP27	matrix metallopeptidase 27
6.982	NM_010686	LAPTM5	lysosomal protein transmembrane 5
6.966	NM_019980	LITAF	lipopolysaccharide-induced TNF factor
6.964	NM_009841	CD14	CD14 molecule
6.927	NM_011332	CCL17	chemokine (C-C motif) ligand 17
6.853	NM_031159	APOBEC1	apolipoprotein B mRNA editing enzyme, catalytic polypeptide 1
6.776	XM_131779	C1orf38	chromosome 1 open reading frame 38
6.768	NM_013674	IRF4	interferon regulatory factor 4
6.699	NM_007464	BIRC3	baculoviral IAP repeat containing 3
6.672	NM_173398	GPR171	G protein-coupled receptor 171
6.604	NM_013563	IL2RG	interleukin 2 receptor, gamma
6.604	NM_010738	Ly6a (includes others)	lymphocyte antigen 6 complex, locus A
6.599	NM_007798	CTSB	cathepsin B

6.444	NM_027222	MZB1	marginal zone B and B1 cell-specific protein
6.364	NM_007895	Ear2 (includes others)	eosinophil-associated, ribonuclease A family, member 2
6.318	NM_146069	LRRC33	leucine rich repeat containing 33
6.315	NM_007609	CASP4	caspase 4, apoptosis-related cysteine peptidase
6.304	NM_010658	MAFB	v-maf musculoaponeurotic fibrosarcoma oncogene homolog B (avian)
6.296	NM_130905	Cd209e	CD209e antigen
6.282	NM_007535	Bcl2a1c	B cell leukemia/lymphoma 2 related protein A1c
6.248	NM_008528	BLNK	B-cell linker
6.234	NM_007646	CD38	CD38 molecule
6.195	NM_009896	SOCS1	suppressor of cytokine signaling 1
6.174	NM_153505	NCKAP1L	NCK-associated protein 1-like
6.165	NM_008879	LCP1	lymphocyte cytosolic protein 1 (L-plastin)
6.157	NM_012057	IRF5	interferon regulatory factor 5
6.119	NM_153074	LRRC25	leucine rich repeat containing 25
6.031	NM_027521	HMHA1	histocompatibility (minor) HA-1
6.011	NM_011723	XDH	xanthine dehydrogenase
6.002	NM_008548	MAN1A1	mannosidase, alpha, class 1A, member 1
5.989	NM_011607	TNC (includes EG:116640)	tenascin C
5.977	NM_023143	C1R	complement component 1, r subcomponent
5.948	NM_029803	IFI27L2	interferon, alpha-inducible protein 27-like 2
5.914	NM_009663	ALOX5AP	arachidonate 5-lipoxygenase-activating protein
5.890	NM_023065	IFI30	interferon, gamma-inducible protein 30
5.739	NM_008677	NCF4	neutrophil cytosolic factor 4, 40kDa
5.675	NM_144835	HEATR1	HEAT repeat containing 1
5.620	NM_008625	MRC1 (includes EG:100286774)	mannose receptor, C type 1
5.597	NM_010380	HLA-C	major histocompatibility complex, class I, C
5.515	NM_011113	PLAUR	plasminogen activator, urokinase receptor
5.484	NM_009163	SGPL1	sphingosine-1-phosphate lyase 1
5.479	NM_144834	SERPINA10	serpin peptidase inhibitor, clade A (alpha-1 antiproteinase, antitrypsin), member 10
5.456	NM_009146	FRRS1	ferric-chelate reductase 1
5.438	NM_024225	SNX5	sorting nexin 5
5.413	NM_011815	FYB	FYN binding protein

5.342	NM_007641	MS4A1	membrane-spanning 4-domains, subfamily A, member 1
5.333	NM_007782	CSF3R	colony stimulating factor 3 receptor (granulocyte)
5.327	NM_007459	AP2A2	adaptor-related protein complex 2, alpha 2 subunit
5.293	NM_024457	RAP1B	RAP1B, member of RAS oncogene family
5.266	NM_028608	GLIPR1	GLI pathogenesis-related 1
5.243	NM_010186	FCGR1A	Fc fragment of IgG, high affinity Ia, receptor (CD64)
5.171	NM_009137	CCL22	chemokine (C-C motif) ligand 22
5.169	NM_009983	CTSD	cathepsin D
5.163	XM_358306	ABCC3	ATP-binding cassette, sub-family C (CFTR/MRP), member 3
5.141	NM_184052	IGF1	insulin-like growth factor 1 (somatomedin C)
5.113	NM_008091	GATA3	GATA binding protein 3
5.093	NM_133871	IFI44	interferon-induced protein 44
5.023	NM_007486	ARHGDIB	Rho GDP dissociation inhibitor (GDI) beta
4.793	NM_009735	B2M	beta-2-microglobulin
4.770	NM_028749	NPL	N-acetylneuraminate pyruvate lyase (dihydrodipicolinate synthase)
4.741	NM_007408	PLIN2	perilipin 2
4.731	NM_013820	HK2	hexokinase 2
4.721	NM_025994	EFHD2	EF-hand domain family, member D2
4.663	NM_009109	RYR1 (includes EG:20190)	ryanodine receptor 1 (skeletal)
4.587	NM_009263	SPP1 (includes EG:20750)	secreted phosphoprotein 1
4.514	NM_146251	PNPLA7	patatin-like phospholipase domain containing 7
4.488	NM_011662	TYROBP	TYRO protein tyrosine kinase binding protein
4.472	NM_010394	HLA-B	major histocompatibility complex, class I, B
4.433	NM_009008	RAC2	ras-related C3 botulinum toxin substrate 2 (rho family, small GTP binding protein Rac2)
4.411	NM_010870	Naip5 (includes others)	NLR family, apoptosis inhibitory protein 1
4.393	NM_010545	CD74	CD74 molecule, major histocompatibility complex, class II invariant chain
4.388	NM_011150	LGALS3BP	lectin, galactoside-binding, soluble, 3 binding protein

4.357	NM_008638	MTHFD2	methylenetetrahydrofolate dehydrogenase (NADP+ dependent) 2, methenyltetrahydrofolate cyclohydrolase
4.347	NM_019753	CDH17	cadherin 17, LI cadherin (liver-intestine)
4.313	NM_153795	FERMT3	fermitin family member 3
4.209	NM_010846	MX1	myxovirus (influenza virus) resistance 1, interferon-inducible protein p78 (mouse)
4.204	NM_019777	IKBKE	inhibitor of kappa light polypeptide gene enhancer in B-cells, kinase epsilon
4.198	NM_010368	GUSB	glucuronidase, beta
4.172	NM_205820	Tlr13	toll-like receptor 13
4.168	NM_133662	IER3	immediate early response 3
4.140	NM_009174	SIAH2	siah E3 ubiquitin protein ligase 2
4.122	NM_025967	C21orf91	chromosome 21 open reading frame 91
4.061	NM_010581	CD47	CD47 molecule
4.059	NM_009787	PDIA4	protein disulfide isomerase family A, member 4
4.059	NM_011893	SH3BP2	SH3-domain binding protein 2
4.040	NM_008390	IRF1 (includes EG:16362)	interferon regulatory factor 1
4.027	NM_022324	SDF2L1	stromal cell-derived factor 2-like 1
3.983	NM_008330	Ifi47	interferon gamma inducible protein 47
3.941	NM_013842	XBP1 (includes EG:140614)	X-box binding protein 1
3.932	NM_011671	UCP2	uncoupling protein 2 (mitochondrial, proton carrier)
3.929	NM_019440	Irgm2	immunity-related GTPase family M member 2
3.916	NM_010130	EMR1	egf-like module containing, mucin-like, hormone receptor-like 1
3.886	NM_007806	CYBA	cytochrome b-245, alpha polypeptide
3.875	NM_023409	NPC2 (includes EG:10577)	Niemann-Pick disease, type C2
3.841	NM_010864	MYO5A	myosin VA (heavy chain 12, myoxin)
3.840	NM_181545	SLFN13	schlafen family member 13
3.730	NM_009121	SAT1	spermidine/spermine N1-acetyltransferase 1
3.681	NM_013585	PSMB9	proteasome (prosome, macropain) subunit, beta type, 9 (large multifunctional peptidase 2)
3.660	NM_007599	CAPG	capping protein (actin filament), gelsolin-like

3.649	NM_007752	CP	ceruloplasmin (ferroxidase)
3.628	NM_010259	Gbp1	guanylate binding protein 1
3.587	NM_010696	LCP2	lymphocyte cytosolic protein 2 (SH2 domain containing leukocyte protein of 76kDa)
3.570	NM_013640	PSMB10	proteasome (prosome, macropain) subunit, beta type, 10
3.570	XM_203404	SKAP1	src kinase associated phosphoprotein 1
3.451	NM_010188	FCGR2A	Fc fragment of IgG, low affinity IIa, receptor (CD32)
3.441	NM_010849	MYC	v-myc myelocytomatosis viral oncogene homolog (avian)
3.412	NM_018738	Igtp	interferon gamma induced GTPase
3.332	NM_007404	ADAM9	ADAM metallopeptidase domain 9
3.331	NM_025378	IFITM3	interferon induced transmembrane protein 3
3.309	NM_018764	PCDH7	protocadherin 7
3.291	NM_009883	CEBPB (includes EG:1051)	CCAAT/enhancer binding protein (C/EBP), beta
3.278	NM_019734	ASAH1	N-acylsphingosine amidohydrolase (acid ceramidase) 1
3.271	NM_016687	SFRP4	secreted frizzled-related protein 4
3.258	NM_010589	JAK3	Janus kinase 3
3.203	NM_010407	HCK	hemopoietic cell kinase
3.190	NM_026929	CHAC1	ChaC, cation transport regulator homolog 1 (E. coli)
3.176	NM_011103	PRKCD	protein kinase C, delta
3.125	NM_007413	ADORA2B	adenosine A2b receptor
3.097	NM_133832	RDH10	retinol dehydrogenase 10 (all-trans)
3.092	NM_008175	GRN	granulin
3.073	NM_010240	FTL	ferritin, light polypeptide
3.058	XM_126809	PDIA6	protein disulfide isomerase family A, member 6
3.044	NM_028727	NOL9	nucleolar protein 9
3.040	NM_021278	TMSB10/TMS B4X	thymosin beta 4, X-linked
3.020	NM_172393	AIM1 (includes	absent in melanoma 1
2.997	NM_146015	EFEMP1	EGF containing fibulin-like extracellular matrix protein 1
2.980	NM_020557	CMPK2	cytidine monophosphate (UMP-CMP) kinase 2, mitochondrial
2.954	NM_008054	FYN	FYN oncogene related to SRC, FGR, YES
2.897	NM_017372	Lyz1/Lyz2	lysozyme 2
2.837	NM_010819	CLEC4D	C-type lectin domain family 4, member D
2.828	NM_025468	SEC11C	SEC11 homolog C (S. cerevisiae)
2.815	NM_028003	RPAP3	RNA polymerase II associated protein 3

2.811	NM_028270	ALDH1B1	aldehyde dehydrogenase 1 family, member B1
2.800	XM_488522	PARP14	poly (ADP-ribose) polymerase family, member 14
2.798	NM_194344	SH3TC1	SH3 domain and tetratricopeptide repeats 1
2.796	XM_486478	LOC434624	ferritin light chain 1-like
2.790	NM_025326	TMEM176A	transmembrane protein 176A
2.705	NM_153783	PAOX	polyamine oxidase (exo-N4-amino)
2.679	NM_025408	ACER3	alkaline ceramidase 3
2.670	NM_133232	PFKFB3	6-phosphofructo-2-kinase/fructose-2,6-biphosphatase 3
2.661	NM_009283	STAT1	signal transducer and activator of transcription 1, 91kDa
2.613	NM_009151	SELPLG	selectin P ligand
2.603	NM_008225	HCLS1	hematopoietic cell-specific Lyn substrate 1
2.566	NM_021389	SH3KBP1	SH3-domain kinase binding protein 1
2.527	NM_013739	DOK3	docking protein 3
2.519	NM_009396	TNFAIP2	tumor necrosis factor, alpha-induced protein 2
2.518	NM_030685	SERP1	stress-associated endoplasmic reticulum protein 1
2.515	NM_011633	TRAF5	TNF receptor-associated factor 5
2.491	XM_125543	TUBE1	tubulin, epsilon 1
2.478	NM_017370	HP	haptoglobin
2.474	NM_011404	SLC7A5	solute carrier family 7 (amino acid transporter light chain, L system), member 5
2.465	NM_133910	TBC1D14	TBC1 domain family, member 14
2.449	NM_013790	ABCC5	ATP-binding cassette, sub-family C (CFTR/MRP), member 5
2.441	NM_172572	RHBDF2	rhomboid 5 homolog 2 (Drosophila)
2.423	NM_198021	SCYL2	SCY1-like 2 (S. cerevisiae)
2.419	NM_028230	SHMT2	serine hydroxymethyltransferase 2 (mitochondrial)
2.384	NM_008407	ITIH3	inter-alpha-trypsin inhibitor heavy chain 3
2.364	NM_144832	C17orf62	chromosome 17 open reading frame 62
2.362	NM_013637	PRM1 (includes EG:19118)	protamine 1
2.351	NM_010459	HOXB4	homeobox B4
2.333	NM_001003918	USP7	ubiquitin specific peptidase 7 (herpes virus-associated)
2.326	XM_133543	AKAP13	A kinase (PRKA) anchor protein 13
2.307	NM_007833	DCN	decorin
2.274	NM_016812	BANP	BTG3 associated nuclear protein
2.251	NM_011072	PFN1	profilin 1

2.238	NM_007454	AP1B1	adaptor-related protein complex 1, beta 1 subunit
2.219	NM_010230	Fmn1	formin 1
2.216	NM_009035	RBPJ	recombination signal binding protein for immunoglobulin kappa J region
2.214	NM_028035	SNX10	sorting nexin 10
2.191	NM_029767	RPS9	ribosomal protein S9
2.181	NM_007465	BIRC2	baculoviral IAP repeat containing 2
2.156	XM_134198	TMEM192	transmembrane protein 192
2.148	NM_027532	C11orf51	chromosome 11 open reading frame 51
2.147	NM_175095	COMMD2	COMM domain containing 2
2.138	NM_028876	TMED5	transmembrane emp24 protein transport domain containing 5
2.127	NM_019663	PIAS1	protein inhibitor of activated STAT, 1
2.127	NM_007644	SCARB2	scavenger receptor class B, member 2
2.105	NM_009729	ATP6V0C	ATPase, H+ transporting, lysosomal 16kDa, V0 subunit c
2.098	NM_011378	SIN3A	SIN3 transcription regulator homolog A (yeast)
2.083	XM_285326	CARD9	caspase recruitment domain family, member 9
2.060	NM_008774	PABPC1	poly(A) binding protein, cytoplasmic 1
2.046	NM_022314	TPM3	tropomyosin 3
2.045	XM_357752	Gm5331	predicted gene 5331
2.042	NM_019642	RPN2	ribophorin II
2.038	NM_134114	SFT2D1	SFT2 domain containing 1
2.033	NM_009796	CAPN7	calpain 7
2.019	NM_025383	NECAP2	NECAP endocytosis associated 2
2.009	NM_008495	LGALS1	lectin, galactoside-binding, soluble, 1
2.001	NM_145502	ERLIN1	ER lipid raft associated 1
-2.012	NM_028137	MSANTD3	Myb/SANT-like DNA-binding domain containing 3
-2.015	NM_010219	FKBP4	FK506 binding protein 4, 59kDa
-2.022	NM_153781	PYGB	phosphorylase, glycogen; brain
-2.026	NM_178926	VMAC	vimentin-type intermediate filament associated coiled-coil protein
-2.033	NM_199306	WDTC1	WD and tetratricopeptide repeats 1
-2.042	NM_016900	CAV2	caveolin 2
-2.048	NM_008988	IGDCC3	immunoglobulin superfamily, DCC subclass, member 3
-2.063	NM_172668	LRP4	low density lipoprotein receptor-related protein 4
-2.065	NM_023605	FBXO9	F-box protein 9
-2.089	NM_194269	MORN2	MORN repeat containing 2
-2.116	NM_146236	TCEAL1	transcription elongation factor A (SII)-like 1
-2.120	NM_010023	ECI1	enoyl-CoA delta isomerase 1

-2.141	NM_023055	SLC9A3R2	solute carrier family 9, subfamily A (NHE3, cation proton antiporter 3), member 3 regulator 2
-2.158	NM_133348	ACOT7	acyl-CoA thioesterase 7
-2.160	NM_175380	GPD1L	glycerol-3-phosphate dehydrogenase 1-like
-2.160	NM_146169	Paip2b	poly(A) binding protein interacting protein 2B
-2.166	NM_025912	FAM210B	family with sequence similarity 210, member B
-2.171	NM_145562	PARM1	prostate androgen-regulated mucin-like protein 1
-2.182	NM_008056	FZD6	frizzled family receptor 6
-2.221	NM_011506	SUCLA2	succinate-CoA ligase, ADP-forming, beta subunit
-2.251	NM_198161	BHLHB9	basic helix-loop-helix domain containing, class B, 9
-2.339	NM_019942	SEPT6	septin 6
-2.356	XM_129968	PHLPP1	PH domain and leucine rich repeat protein phosphatase 1
-2.357	NM_009166	SORBS1	sorbin and SH3 domain containing 1
-2.364	NM_145441	UBXN2A	UBX domain protein 2A
-2.380	NM_009685	APBB1	amyloid beta (A4) precursor protein-binding, family B, member 1 (Fe65)
-2.388	XM_129894	ACSL3	acyl-CoA synthetase long-chain family member 3
-2.394	NM_011698	LIN7B	lin-7 homolog B (C. elegans)
-2.395	NM_175279	RASSF10	Ras association (RalGDS/AF-6) domain family (N-terminal) member 10
-2.406	NM_022433	SIRT3	sirtuin 3
-2.412	NM_007929	EMP2	epithelial membrane protein 2
-2.443	NM_175126	ZCCHC3	zinc finger, CCHC domain containing 3
-2.466	NM_023824	PAQR4	progestin and adipoQ receptor family member IV
-2.501	NM_019656	TSPAN6	tetraspanin 6
-2.510	NM_008973	PTN	pleiotrophin
-2.515	NM_031177	IFT122	intraflagellar transport 122 homolog (Chlamydomonas)
-2.534	NM_181588	CMBL	carboxymethylenebutenolidase homolog (Pseudomonas)
-2.598	NM_145144	AIF1L	allograft inflammatory factor 1-like
-2.650	NM_130448	PCDH18	protocadherin 18
-2.653	NM_015814	DKK3	dickkopf 3 homolog (Xenopus laevis)
-2.678	NM_029850	BCL7A	B-cell CLL/lymphoma 7A
-2.760	NM_176848	FBXO2	F-box protein 2
-2.777	NM_011670	UCHL1	ubiquitin carboxyl-terminal esterase L1 (ubiquitin thiolesterase)
-2.836	NM_010762	MAL	mal, T-cell differentiation protein
-2.852	NM_011448	SOX9	SRY (sex determining region Y)-box 9

-2.893	NM_145463	SHISA2	shisa homolog 2 (Xenopus laevis)
-2.945	NM_019410	PFN2	profilin 2
-3.031	NM_178929	KAZALD1	Kazal-type serine peptidase inhibitor domain 1
-3.076	NM_019814	Higd1a	HIG1 domain family, member 1A
-3.104	NM_178921	PRLR	prolactin receptor
-3.132	XM_194040	MAP1A	microtubule-associated protein 1A
-3.134	NM_013509	ENO2	enolase 2 (gamma, neuronal)
-3.137	NM_007736	COL4A5	collagen, type IV, alpha 5
-3.144	NM_008135	SLC6A9 (includes EG:116509)	solute carrier family 6 (neurotransmitter transporter, glycine), member 9
-3.177	NM_175666	HIST2H2BF	histone cluster 2, H2bf
-3.378	NM_030706	TRIM2	tripartite motif containing 2
-3.432	NM_026754	UCMA	upper zone of growth plate and cartilage matrix associated
-3.552	NM_053078	NREP	neuronal regeneration related protein homolog (rat)
-3.570	XM_125798	BTBD11	BTB (POZ) domain containing 11
-3.587	NM_001001981	Utp14b	UTP14, U3 small nucleolar ribonucleoprotein, homolog B (yeast)
-3.598	NM_009368	TGFB3	transforming growth factor, beta 3
-3.660	NM_013467	ALDH1A1	aldehyde dehydrogenase 1 family, member A1
-3.736	NM_009144	SFRP2	secreted frizzled-related protein 2
-3.737	NM_198108	MORN4	MORN repeat containing 4
-3.790	NM_027990	LYPD6B	LY6/PLAUR domain containing 6B
-3.829	NM_172399	NDNF	neuron-derived neurotrophic factor
-3.830	XM_355606	RASL11B	RAS-like, family 11, member B
-3.836	NM_025449	NICN1	nicolin 1
-3.847	NM_144807	CHPT1	choline phosphotransferase 1
-3.860	XM_283704	ENKUR	enkurin, TRPC channel interacting protein
-3.866	NM_009221	SNCA	synuclein, alpha (non A4 component of amyloid precursor)
-3.921	XM_288123	ARHGEF9	Cdc42 guanine nucleotide exchange factor (GEF) 9
-3.935	NM_030262	POFUT2	protein O-fucosyltransferase 2
-3.947	NM_011438	SOX12	SRY (sex determining region Y)-box 12
-4.052	NM_029916	STK31	serine/threonine kinase 31
-4.305	NM_146054	FERMT2	fermitin family member 2
-4.362	NM_017400	SH3GL3	SH3-domain GRB2-like 3
-4.544	NM_054068	VSX1	visual system homeobox 1
-4.601	NM_001001979	MEGF10	multiple EGF-like-domains 10
-4.716	NM_013743	PDK4	pyruvate dehydrogenase kinase, isozyme 4
-4.944	XM_128418	PACRG	PARK2 co-regulated
-4.993	NM_021272	FABP7	fatty acid binding protein 7, brain
-5.114	NM_008747	NTSR2	neurotensin receptor 2

-5.336	NM_008778	PAK3	p21 protein (Cdc42/Rac)-activated kinase 3
-5.502	NM_183315	CTXN1	cortexin 1
-6.702	NM_011784	APLNR	apelin receptor
-6.868	NM_145602	NDRG4	NDRG family member 4
-7.184	NM_010883	NDP	Norrie disease (pseudoglioma)
-7.450	NM_022029	NRGN	neurogranin (protein kinase C substrate, RC3)
-9.392	NM_007541	Bglap (includes others)	bone gamma carboxyglutamate protein
-12.020	NM_198637	C17orf97	chromosome 17 open reading frame 97
-12.340	NM_008165	GRIA1	glutamate receptor, ionotropic, AMPA 1
-14.120	NM_198119	LRRC24	leucine rich repeat containing 24
-18.700	NM_177624	SNTN	sentan, cilia apical structure protein
-43.860	NM_029530	LAMP5	lysosomal-associated membrane protein family, member 5
