# Supplementary material for: Changes in Gene Expression of Pial Vessels of the Blood Brain Barrier during Murine Neurocysticercosis
Source: PLoS Negl Trop Dis. 2013 Mar 14;7(3):e2099. doi: 10.1371/journal.pntd.0002099 (PMC3597490; doi:10.1371/journal.pntd.0002099)
Supplement: Table S2 — List of significant networks of genes. Networks associated with differentially expressed genes in pial endothelial cells containing down regulated genes shown by green color, upregulated genes shown by red color and genes which are not affected in endothelial cells during infection but relevant for the generation of the networks are shown in black color. (PDF) [file pntd.0002099.s002.pdf]

ID	Molecules in Network	Score	Focus Molecule	Top Functions
1	**ACP5**, **AKAP13**, **ATP6V0D2**, **C3**, **CCL5**, **CCL7**, **CCL22**, **CD14**, **CFP**, chemokine, Ctbp, **CXCL3**, **CXCL13**, **CXCR4**, **EMP2**, **FRRS1**, hCG, **HK2**, **HPSE**, **IER3**, Ikb, **IKBKE**, IL1, IL12 (family), **IRF4**, **Lyz1/Lyz2**, **MSR1 (includes EG:20288)**, NfkB1-RelA, **PDIA6**, **PFKFB3**, **SLFN12L**, Tlr, **TMSB10/TMSB4X**, **TNFSF11**, Vegf	42	26	Inflammatory response, cell-to-cell signaling and interaction, cellular movement
2	**ABCC3**, **ADA**, **BST1 (includes EG:12182)**, **C6**, C8, **Ccl6**, **Ccl9**, **CCL13**, **CCL17**, **CD74**, chitinase, chymotrypsin, collagen, **CTSL2, CTSS, CXCL2,** elastase, **GRN**, **IL1R2**, immune, **LGMN**, Mapk, Mhc class ii, MHC Class II (complex), **MMP12**, **PARP14**, PARP, **PLAUR**, **SELP**, **SELPLG**, **SLPI**, Tnf, Tnf receptor, **UBD**, **UCHL1**	37	23	Cellular movement, hematological system development and function, immune cell trafficking
3	**26s Proteasome**, **AP2A2**, **APBB1**, **BIRC2**, **BIRC3**, calpain, **CAPN7**, **CASP4**, caspase, **CCL2**, Cdk, **CMPK2**, **CP**, Cyclin D, Cyclin E, Cytochrome c, **FTL**, Hsp27, **IGDCC3**, LDL, **LRP4**, LRP, Mek, **Naip5 (includes others)**, **NPC2 (includes EG:10577)**, **PABPC1**, **Pmaip1**, **PTN**, Rb, **RYR1 (includes EG:20190)**, **SAT1**, **SLFN13**, **STAT1**, **TIMP1**, trypsin	34	22	Antimicrobial response, cell-to-cell signaling and interaction, embryonic development
4	**ADAM9**, Akt,B-cell receptor, **BLNK**, **CD52**, **CYTIP**, Fc gamma receptor, Fcer1, FCGR1A/2A/3A, **FCGR2A**, **FCGR2B**, **FERMT2**, Fgfr, **FYB**, GAD, **HCLS1**, **HCST**, **Igtp**, Integrinβ, **LCP2**, LDL-cholesterol, Lfa-1, **LPXN**, **MAFB**, NCK, **PLAC8**, PLC gamma, **SH3BP2**, **SIRT3**, **SKAP1**, SYK/ZAP, **TREM2**, **TYROBP**, **VAV1**, VAV	32	21	Hematological system development and function, tissue morphology, cell cycle
5	**ALOX5AP**, **ARG1**, arginase, **C1orf38**, **CSF3R**, **CTSZ**, **CYBA**, ERK, Fc eceptor, **FCER1G**, **FCGR1A**, **FERMT3**, Gm-csf, **HCK**, HLA-DR, **IFI44L**, Ifn gamma, IL23, Interferon-α Induced, **IRF1 (includes EG:16362)**, **LGALS1**, **MAL**, NADPH oxidase, **NCF4**, Oas, **PCDH7**, **PDK4**, Rock, **SGPL1**, **SOCS3**, Stat1 dimer, STAT5a/b, TH1 Cytokine, TH2 Cytokine, **TMEM173**	30	20	Connective tissue disorders, inflammatory disease, skeletal and muscular disorders
6	Actin, Adaptor protein 1, alcohol group acceptor phosphotransferase, Alpha tubulin, Ap1 gamma, **AP1B1**, **ARHGDIB**, Arp2/3, **CARD9**, Caspase 3/7, **CTSD**, Dynamin, Endophilin, Hd-perinuclear inclusions, Hsp70, Jnk, **MAP1A**, **MZB1**, **PACRG**, **PAK3**, Pak, **PFN1**, **PFN2**, **PIAS1**, Profilin, **SCYL2**, **SFRP2**, **SFRP4**, SFRP, **SH3GL3**, **SH3KBP1**, **SHMT2**, **SIAH2**, **SNCA**, **TFRC**	30	20	Cellular assembly and organization, cellular function and maintenance, protein synthesis
7	**ARHGEF9**, Cbp/p300, **CD47**, **CEBPB (includes EG:1051)**, **CHPT1**, **Creb**, **GATA3**, Gcn5l, **HAL**, Hdac, hemoglobin, HISTONE, Histone h3, Histone h4, **HOXB4**, **Iigp1/Iigp1b**, IL12 (complex), Immunoglobulin, **Irgm2**, **Ms4a4b (includes others)**, **NDRG4**, P38 MAPK, Pias, Pro-inflammatory Cytokine, **RAP1B**, **RBPJ**, **SEC11C**, **SIN3A**, **SOCS1**, **SOX9**, **SOX12**, **SPP1 (includes EG:20750)**, **TMEM176A**, TSH, **XBP1 (includes EG:140614)**	30	21	Cellular development, digestives development and function, embryonic development

8	**ALDH1B1**, **C1R**, Cbp (family), Ciap, **CST7**, **DOK3**, **Gbp1**, **IFI30**, **IFI44**, **Ifi47**, **Ifi202b**, **Ifi204 (includes others)**, **IFI27L2**, **IFITM3**, Ifn, IFN TYPE 1, IRAK, **IRF5**, IRF, IRG, **LITAF**, lymphotoxin, lymphotoxin-alpha1-beta2, **MX1**, NFkB (complex), NfkB-RelA, **OAS2**, peptidase, SOCS, Stat3-Stat3, **TICAM2**, **Tlr13**, **TNFAIP2**, Vacuolar H+ ATPase, **ZBP1**	29	20	Dermatological diseases and conditions, immunological disease, inflammatory disease
9	**ACER3**, **ASAH1**, BCR (complex), **CD38**, **CDH17**, Cyclin A, **DKK3**, E2f, **ENO2**, **Fcgr2**, **Gp49a/Lilrb4**, **Higd1a**, Iga, Ige, IgG1, Igg3, IgG, Igh (family), Igm, **LAPTM5**, **LGALS3**, PI3K (family), PI3K p85, **PIM1**, Pld, **PLEK**, **PRKCD**, Ras, Rsk, Sapk, **SDF2L1**, **SIGLEC1**, **SLC7A5**, **TMED5**, **TRAF5**	26	18	Humoral immune response, protein synthesis, lipid metabolism
10	ALDH4A1, **Ang2 (includes others)**, AP3B1, APOH, ARRB1, **ATP6V0C**, beta-estradiol, C4B (includes others), **CLEC4D**, **CMBL**, **COMMD2**, CXCL16, **FASTKD3**, **GALNT6**, **GLIPR1**, Gm11428, **GPNMB**, **IFI30**, IFNG (includes EG:15978), **MEGF10,** NUB1, P2RY6, **RAB20**, RELA, **SAA1**, SCGB1A1, **SLAMF6**, **SLC15A3**, **SLPI**, SNRNP200, **SNX5**, TAF9, TP53 (includes EG:22059), VHL, **VSX1**	25	18	Gene expression, infectious disease, immunological disease
11	**ABCC5, ACOT7,** ACOT8, ACOT9, ACOT13, **AKR1C3, BCL7A,** BRK1, CDC42EP3, **CHAC1, ECI1, ENKUR, ERLIN1,** GNPAT, **IFT122,** IFT140, **NCKAP1L, NECAP2,** PHAX, PPT1, **PYGB, PYGL, RDH10,** SEPT1, SEPT3, SEPT4, **SEPT6,** SEPT8, SEPT11, **SNX10,** TULP3, UBC, **UBXN2A, UBXN2B, WDTC1**	24	17	Cell morphology, cellular compromise, carbohydrate metabolism
12	20s proteasome, **B2M,** beta-glucuronidase, **CAPG,** CD8, **CD274, GUSB,** HLA Class I, Hla-abc, **HLA-B, HLA-C,** IFN alpha/beta, IFN Beta, Ifnar, IgG2a, IL-2R, IL17R, **IL2RG,** INTERLEUKIN, **JAK3,** JAK, **Ly6a (includes others), MAN1A1,** Mannosidase Alpha, MHC, MHC Class I (complex), MHC CLASS I (family), MHC I-α, **MRC1 (includes EG:100286774), MTHFD2, PDCD1LG2 (includes EG:309304),** PI3K (complex), **PSMB10, SAMHD1, SASH3**	22	16	Cell morphology, hematological system development and function, tissue morphology
13	**AIF1L, BHLHB9, C17orf62,** COX5A (includes EG:12858), DUSP6, **EFHD2, FAM210B,** FGF1, GLG1 (includes EG:20340), GNL3, **GPD1L, HIST2H2BF,** MRPL44 (includes EG:301552), **MSANTD3, NDNF,** PAN2, PCBP3, **POFUT2, RHBDF2,** SDC2, **SFT2D1, SIAH2,** TAF6L, **TMEM192,** UBC, **UCHL1,** UCHL3, USP8, USP13, USP14, USP22, USP28, USP33, USP53, **ZCCHC3**	22	16	Post-translational modification, neurological disease, cardiovascular system development and function
14	2' 5' oas, **ABCG1**, **ACSL3**, **AKR1C3**, **APOBEC1**, C/ebp, Cebp, CK1, ERK1/2, Ferritin, **FGA**, HDL, **HP**, **ITIH4**, JINK1/2, Mac1, N-cor, Nr1h, **NRGN**, PEPCK, **PHLPP1**, **PLIN2**, PTPase, Rxr, **SAA1**, **SAA2**, SAA, **Slfn1**, SWI-SNF, T3-TR-RXR, thymidine kinase, **TRIM2**, **UCP2**, VitaminD3-VDR-RXR, **XDH**	22	16	Lipid metabolism, molecular transport, small molecule biochemistry
15	Adaptor protein 2, ADCY, **ADORA2B,** ADRB, Beta Arrestin, **C3**, C3AR1, Ck2, ENTPD1, **FBXO2**, **FKBP4**, FSH, GALR3, GPR77, Gsk3, **HEATR1**, **HMOX1**, HRH3, **Ifi204 (includes others)**, IKK (complex), Interferon alpha, **LGMN**, LTB4R, MC3R, Metalloprotease, **MYC**, **NOL9**, **NREP**, **PSMB9**, RNA polymerase II, **RPS9**, **SLC9A3R2**, STAT, **SUCLA2**, Ubiquitin	19	15	Cellular compromise, inflammatory response, inflammatory disease

16	**AIM1 (includes EG:11630)**, **APLNR**, APOH, BDNF, C2, **C11orf51**, C1S, CCL23, CCR10, CX3CR1, CXCR5, **Ear2 (includes others)**, **Fcna**, HNF4A, **IFI30**, **ITIH3**, **ITIH4**, **LAMP5**, **LRRC25**, LSR, Mac1, MAPK1, MMP24, **MMP27**, Mmp, **MS4A8B**, POSTN, **SAA1**, **SERPINA10,** TGFB1 (includes EG:21803), Timp, TLR2, tretinoin, **TUBE1**, ULBP3	19	15	Cellular movement, cell signaling, molecular transport
17	Alp, Alpha catenin, **BANP,** C1q, **CAV2, CD68,** Collagen Alpha1, Collagen type I, Collagen type IV, Collagen(s), Complement component 1, **CTSB, DCN,** Fibrin, Fibrinogen, GPIIB-IIIA, IgG2b, Integrin alpha V beta 3, **LAIR1,** Laminin1, Laminin, **MS4A1,** Pdgf (complex), PDGF BB, **PDIA4,** Pkc(s), Ppp2c, **REG3A, Retnla, RPN2, SLC6A9 (includes EG:116509), SLFN12,** Tgf beta, **TGFB3, TNC (includes EG:116640)**	18	15	Lipid metabolism, molecular transport, small molecule biochemistry
18	14-3-3, **AGPAT9, Bglap (includes others),** Calcineurin protein(s), Calmodulin, CaMKII, Cg, **CTXN1,** Ecm, **EFEMP1,** F Actin, **GRIA1,** Growth hormone, Hsp90, Insulin, ITPR, **LCP1, LGALS3BP**, Lh, **LIN7B,** MAP2K1/2, **MYO5A,** NFAT (complex), Nfat (family), p70 S6k, Pka, PP2A, **PRLR,** Proinsulin, **RPAP3, SERP1, SORBS1, TPM3,** Tropomyosin, **Utp14b**	17	15	Endocrine system development and function, organ morphology, organismal development
19	**BTBD11**, **C21orf91**, C3AR1, **CCKAR**, CCR10, CCRL1, CX3CR1, DLG4, **EMR1**, FZD4, **FZD6**, GALR3, Gpcr, **GPR18**, GPR45, GPR63, GPR68, GPR77, **GPR171**, GRB2, HCAR3, HRH3, HTR5A, LTB4R, MC3R, **NDP**, **NPL**, **NTSR2**, P2RY6, **PARM1**, progesterone, Relaxin, **RGS1**, UTS2R, **VMAC**	17	13	Cell signaling, molecular transport, vitamin and mineral metabolism
20	ADIPOR1, **ALDH1A1**, **ALDH1B1**, ALDH4A1, AQP9, **B3GNT6**, CLIC2, D-glucose, EZH2, **FBXO9**, GLRX, HSDL2, LGALSL, **LYPD6B**, MAGEE1, NEDD4, **PNPLA7**, POR, PRKAG3, **RASL11B**, **SAA1**, SELS, **SHISA2**, SLC25A36, **STK31**, STT3A, **TBC1D14**, TBCB, **TCEAL1**, TOM1L2, TSPYL5, UBC, **USP7**, USP53, YY1	17	13	Drug metabolism, lipid metabolism, molecular transport
21	AMPK, Ap1, CD3, **COL4A5**, estrogen receptor, **Fmn1**, Focal adhesion kinase, **FYN**, G protein, G protein alphai, G-protein beta, Gpcr, **IGF1**, Integrin, Mlc, Mmp, **OASL**, p85 (pik3r), **PADI4**, **PAOX**, **PCDH18**, Pdgfr, PLA2, PLC, **RAC2**, Rac, Ras homolog, **SCARB2**, Sfk, Shc, Sos, SRC (family), **SRGN**,TCR, Tubulin	11	11	Cell cycle, hair and skin development and function, cellular function and maintenance
22	ALB, ASNS, choline, COL18A1, **CTSL2**, DDX5, ERCC1, FABP3, **FABP7**, FOSB, GNA13, **GRN**, GTF3C2, HRAS, **IER3**, IGF2, IGFBP4, Kallikrein, KLF5, MHC, MIR17HG, MNT, p70 S6k, **PCDHGA1**, POU3F2, **PRM1 (includes EG:19118)**, RPS10, RRM2, SH2B2, SPHK1, SPRY1, TBP, **TIMP1**, **TSPAN6**, ULK2	8	8	Cancer, cellular growth and proliferation, cellular development
